# Identifying potential training factors in a vibrotactile P300-BCI

**DOI:** 10.1038/s41598-022-18088-w

**Published:** 2022-08-17

**Authors:** M. Eidel, A. Kübler

**Affiliations:** Institute of Psychology, University of Würzburg, Würzburg, Germany

**Keywords:** Touch receptors, Neural decoding, Machine learning, Electroencephalography - EEG, Brain-machine interface

## Abstract

Brain–computer interfaces (BCI) often rely on visual stimulation and feedback. Potential end-users with impaired vision, however, cannot use these BCIs efficiently and require a non-visual alternative. Both auditory and tactile paradigms have been developed but are often not sufficiently fast or accurate. Thus, it is particularly relevant to investigate if and how users can train and improve performance. We report data from 29 healthy participants who trained with a 4-choice tactile P300-BCI during five sessions. To identify potential training factors, we pre-post assessed the robustness of the BCI performance against increased workload in a dual task condition and determined the participants’ somatosensory sensitivity thresholds with a forced-choice intensity discrimination task. Accuracy (*M* = 79.2% to 92.0%) and tactually evoked P300 amplitudes increased significantly, confirming successful training. Pre-post somatosensory sensitivity increased, and workload decreased significantly, but results of the dual task condition remained inconclusive. The present study confirmed the previously reported feasibility and trainability of our tactile BCI paradigm within a multi-session design. Importantly, we provide first evidence of improvement in the somatosensory system as a potential mediator for the observed training effects.

## Introduction

Brain–computer interfaces (BCIs) allow their users to interact with the external world without the need of any physical movement. This is achieved by acquiring neurophysiological brain activity, mostly non-invasively with electroencephalography (EEG), which is then digitized and interpreted by the computer to control a variety of applications for communication, prosthetic movement or even locomotion^1,2^.

To determine the user’s intent, BCIs often rely on event-related potentials (ERP), such as the P300^3,4^. The P300 can be evoked using variations of the oddball-paradigm^5^. Here, the participants focus their attention on rare relevant stimuli (the targets) appearing randomly within other, frequent stimuli, which have to be ignored (the non-targets). The P300 then occurs as a positive deflection in the EEG at about 300 ms after onset of the target stimuli^6^. Thus, this ERP can be instrumentalized to automatically detect on which stimulus the users had focused their attention. The P300 is usually delineated at electrode positions Fz, Cz and Pz and sometimes divided into its subcomponents P3a and P3b^7^.

Since BCIs are based on brain activity, intact motor function is not required for their successful operation. This independence from voluntary muscular control renders BCI particularly relevant for patients with severe or even complete paralysis^8–11^. Such potential end-users include patients diagnosed with the *locked-in syndrome* (LIS), which can occur due to *amyotrophic lateral sclerosis* (ALS) and numerous other causes^12,13^.

However, when vision or gaze control are impaired, P300 BCIs that depend on visual stimulation can no longer be operated reliably by the end-user^14–16^. Because of this limitation of the prevalent visual BCIs, many paradigms based on the auditory or tactile modalities have been developed. Still, the issue remains that non-visual BCIs are more challenging and workload-intensive than visual paradigms^17^, often resulting in lower BCI performances^18–20^.

Regardless of these limitations, non-visual BCIs have repeatedly been shown to be feasible^18,21–26^. Early tactile BCIs, for instance, were designed to apply stimulation using a waist belt with up to six vibrotactile actuators, which resulted in mean classification accuracies (i.e. percentage of correct selections) of 58%^26^. Others used *braille* stimulators on the fingertips, allowing healthy participants (*N* = 12) to spell short words, and achieved mean accuracies of 67%^27^. Importantly, a few studies have already demonstrated that tactile BCIs can be successfully operated even by severely impaired, potential end-users in the LIS due to stroke or ALS^20,28,29^.

The present study is based on a tactile BCI that was originally intended for virtual wheelchair control and has since repeatedly been demonstrated to be feasible among healthy participants^23,30^, even for the elderly (aged 50–73 years)^31^. Vibrotactile stimulation was applied at four body positions which corresponded to movement commands (front, back, left and right). Over the course of five sessions, significant positive effects on the P300 amplitudes and ITRs were found. While a multi-session training is usually not necessary for visual P300 paradigms, similar training-induced improvements have previously been observed in BCIs based on auditory stimulation^19,25^. It has even been demonstrated that BCI competence acquired from training with an auditory paradigm can, to a degree, be transferred to the tactile modality^32^.

Generally, training effects are not novel in the wide field of BCI. There are self-paced paradigms based on motor imagery, for instance, which the user controls proactively and without external stimulation^33,34^. Here, the user is trained to modulate this signal voluntarily by means of an extensive reinforcement training program, in which feedback of the signal (e.g. the sensorimotor rhythm power) is continuously provided. The underlying mechanisms of these approaches are relatively well understood and apply even for impaired users^35–37^.

Conversely, the cause of performance increases in tactile and auditory P300 BCIs is still poorly understood, and more research is needed to understand how to train end-users and eventually patients to use the BCI efficiently. It is still unclear which factors or training mechanisms are responsible for the observed improvement, specifically within the present tactile paradigm. For this reason, several possibilities should be explored.

Firstly, there is ample support for the idea that training leads to an improvement in the participants’ sensitivity of somatosensory perception. This may facilitate target/non-target discrimination, which is an important factor to elicit the P300^38^. Sensitivity increases due to training have been reported by several non-BCI studies. For instance, Nagarajan et al. revealed that participants had become substantially more sensitive after training on a vibrotactile interval discrimination task over 10–15 days^39^. In line with this result, Imai et al. found that over 30 days of frequency discrimination training, participants became continuously better at the task – most substantially so over the first two weeks^40^. Further, it appears that somatosensory training leads to an enlargement in the sensorimotor cortical areas, which represent the trained body part^41^. Physiological changes due to training might also occur much earlier, at any point along the somatosensory pathways, starting from the actual mechanoreceptors (for an overview on somatosensory pathways, see^42,43^). *Pacinian Corpuscles*, a receptor type in the mammalian skin, may be particularly relevant for tactile BCIs, since they are most sensitive for typical vibrotactile stimuli around 250 Hz^44^. It is known, however, that these receptors decline with age^45^, which should be kept in mind when planning to work with elderly participants. On the other hand, there are several studies which found no evidence for a negative effect of advanced age on tactile BCI performance^31,46,47^.

Secondly, some studies suggested that cognitive factors, e.g. the reduction of mental workload, may be responsible for training-induced performance improvement. In fact, a negative correlation between high workload and BCI performance has been repeatedly demonstrated. For instance, experimenters added additional tasks in parallel to BCI operation to increase mental workload (often referred to as *dual task* condition). In a study by Käthner et al., participants had to spell short words using a visual P300 spelling matrix while also performing a dichotic listening task of medium or high difficulty^48^. Due to the hence increased workload, P300 amplitudes and classification performances were significantly decreased during the dual task. Similar effects were found in a tactile BCI study: Thurlings et al. added a visual monitoring task simultaneously, which resulted in a reduction of P300 amplitudes and ITRs^49^.

Furthermore, BCI training appears to lead to changes in brain activation patterns. To investigate the neural mechanism responsible for training effects, Halder et al. implemented a five session BCI training with an auditory BCI (*N* = 10, healthy)^17^. Functional magnetic resonance imaging (fMRI) data, recorded from the first and last session, revealed that the training had caused an activity decrease in the *superior frontal gyrus* and an increase in the *superior temporal* and *supramarginal gyri*. This observation was attributed to improved stimulus perception and an overall reduction of mental workload. This is in line with several other fMRI studies, which described a reorganization of cortical networks that manifest in different activation patterns during the repeated training of a novel task. Often, the task at first elicits a widespread pattern of cortical activity, which in the training process becomes more focused on areas related to attention and information retrieval^50^. Evidence for this is available from BCI unrelated studies, for instance the training of bimanual coordination, mirror reading or visual search tasks^51–53^.

Such observations are frequently interpreted in the context of Logan’s *instance theory of automatization*^54,55^ which offers a possible mechanism for workload reduction with training. This model describes *automatization* of a novel task as the gradual transfer of an initially workload intensive mental algorithm into a solution that mostly relies on memory retrieval. According to the model, a memory representation (called *instance*) of the solution is generated and consolidated with every task repetition, as long as the task is performed under the same conditions. Driven by training, the mental algorithm for the performance of a task shifts toward an acquired solution guided primarily by memory retrieval, which is potentially more efficient than the initial workload intensive approach^54^. According to the model, the highest training effects are observed early in the learning process. After sufficient training, this *automatization* could eventually even entirely substitute the original algorithm. This in turn would have the effect that much of the initially required attentional resources would become available for other tasks. Typically, *automatization* results in an asymptotic learning curve that adheres to the *power law of practice*^55^.

Information on the neural and physiological mechanisms involved in BCI training remains scarce, especially for tactile paradigms. Thus, the present study was designed not only to replicate and further confirm the trainability of the tactile BCI, but also to contribute to a better understanding of the psychophysiological mechanisms involved in improved BCI performance, such that the paradigm may be further optimized in the future.

Based on numerous previous observations, we hypothesized that P300 amplitudes and differences between target and non-target epochs (H1a/H1b) and BCI accuracies (H2) would increase across five training sessions.

Moreover, we hypothesized that with training, less attentional resources would be required due to automatization. Hence, we expected to find a decreasing mental workload across sessions (H3a) and a negative correlation between workload and BCI performance (H3b). As another indicator of automatization, we hypothesized that BCI performance would become more robust against the negative effects of workload increases during a dual task condition (H3c). Finally, the development of BCI accuracy across sessions was expected to adhere to a power function (H3d).

With regard to somatosensory perception as another potential training factor, we expected a pre-post increase of sensitivity for tactile stimulation (H4a). We also hypothesized that tactile sensitivity would correlate with BCI performance (H4b).

## Methods

### Participants

We recruited *N* = 32 healthy participants to attend a total of five training sessions on separate days, with no more than one week between consecutive sessions. One participant could not complete all sessions due to COVID-19 related precautions. All participants were naïve with respect to BCI operation. They either received a monetary compensation of € 7.50/h or course credits. Participants gave written informed consent to the procedure. The experimental protocol was approved by the Ethics Committee of the Psychological Institute of the University of Würzburg, Germany (approval number: GZEK 2013–11). The study was conducted in accordance with the ethical guidelines of the Declaration of Helsinki.

### EEG recording and processing

EEG data were recorded from twelve passive Ag/AgCl electrodes at a sampling rate of 512 Hz. Electrodes were placed at positions Fz, FC1, FC2, C3, Cz, C4, P3, Pz, P4, O1, Oz, and O2^30^. Ground and reference electrodes were placed at the right and left mastoids, respectively. Impedances for all electrodes were kept below 5 kΩ. The signal was amplified with a g.USBamp (g.tec Engineering GmbH, Graz, Austria). Online filtering was performed using a band pass filter (0.1 to 60 Hz) and a notch filter (48 to 52 Hz).

Further processing steps for offline analysis of the EEG data included band pass filtering (0.1 to 30 Hz) and segmentation into epochs of 800 ms post-stimulus, with an additional 100 ms pre-stimulus period for baseline-correction. Any segments which contained values exceeding ± 100 μV were rejected. The epochs were split into target and non-target groups, which were averaged separately. Analysis was performed with MATLAB© (R2015b) using functions provided by BCI2000^56^. Classifier weights were calculated using the stepwise linear discriminant approach (SWLDA) provided by BCI2000.

### Stimulation

**Figure 1:**
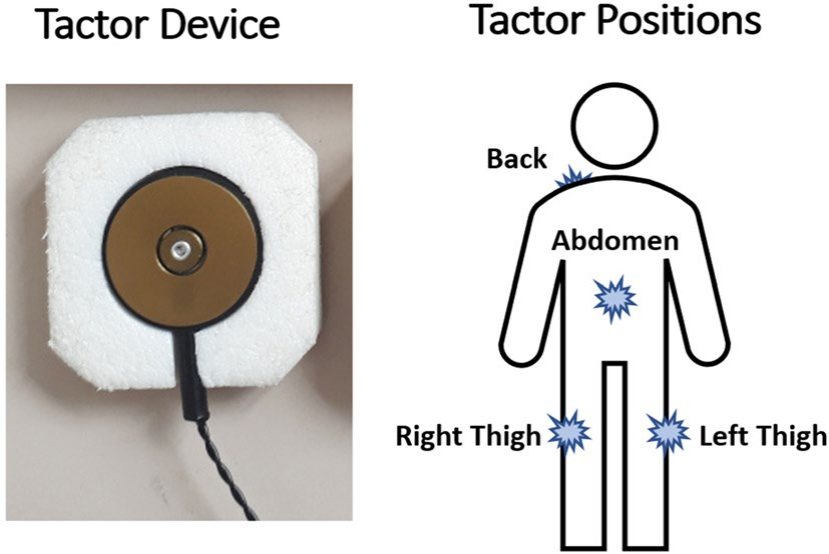
A C2 tactor (by Engineering Acoustic Inc., Casselberry, USA) and the four stimulus positions.

Tactile stimulation was applied at right and left thigh, abdomen (1–5 cm above the navel) and lower neck (approx. at the height of C4–Th3) with BCI2000-controlled vibrotactor devices (C2 tactors; Engineering Acoustic Inc., Casselberry, USA; Fig. 1). The stimulus positions were chosen such that they could be easily memorized and associated with the four directions of virtual wheelchair movement (left/right, front/back; Fig. 1). During a BCI run, these tactor positions vibrated for 220 ms (vibration frequency: 250 Hz) in a pseudorandomized order, with an inter-stimulus interval of 400 ms.

### General session procedure

Participants were seated in front of a monitor displaying the current target tactor position on which they had to concentrate during the BCI tasks. They were advised to avoid blinking and unnecessary movements or muscle activity during recording.

After EEG preparation, three calibration runs were performed with a short pause in between. This was done at the beginning of every session to account for possible training effects^57,58^. For calibration, participants were instructed to concentrate on the vibration at determined target positions (either front, back, left or right) by silently counting how often the current position was activated. Other non-target stimulations had to be ignored. This process resulted in a total of 240 target and 720 non-target trials, which were used to train a linear classifier. After that, the classifier served to provide online feedback during two different BCI tasks: The *copy* and the *dual* task, which are detailed in the next sections.

At the end of each session, participants filled in the NASA-TLX questionnaire^59^ to assess subjective workload.

### Copy task

During the copy task, participants were instructed on which position to concentrate, similar to the preceding calibration runs. In this task however, the classification results were provided as immediate feedback on the monitor and were recorded as a measure of BCI accuracy. Every classification was based on eight sequences (i.e. every tactor position was activated eight times), as this number had been shown to be sufficient in previous studies of the same paradigm^30,31^. For each copy run, eight direction commands had to be given by concentrating on the tactor position indicated on the monitor. The order of the commands was pseudorandomized, such that each position was used twice as the target without immediate repetitions. After three copy tasks, this process resulted in a total of 24 classified commands every session.

### Dual task (session 1 and 5 only)

We included three additional dual task runs to test whether BCI performance would become more robust against the hence increased workload. This may indicate automatization, specifically that more attentional resources were available for the BCI task after training. In the dual task, participants had to listen to a narrated story which was presented via speakers, while also performing a BCI task otherwise identical to the copy task. We used two different excerpts from *Arabian Nights,* recorded from a professional narrator. A similar workload manipulation based on this audiobook had been successfully used before in the BCI context^48^. For the present study, the audio was sped up by a factor of 1.25 since pre-experiments had indicated that the normal speed had not been challenging enough to cause a notable effect. After each run, participants had to answer three open questions about details from the story to ensure and quantify task compliance. The order of the tasks as well as the order of the stories were balanced between participants and sessions, such that not all participants started with the same task and story.

### Attentional resources and automatization

Three potential indicators of automatization were analyzed: Firstly, we assumed that the dual task would cause a workload increase and, hence, a negative impact on BCI performance. To describe this effect, the impact was quantified as follows: Accuracy decreases caused by the dual task were expressed relative to the copy task, resulting in a factor describing the relative accuracy difference between the tasks (Equation 1, P = accuracy).

1 $${\Delta }_{Relative }=\frac{{P}_{dual}-{P}_{copy}}{{P}_{copy}}$$

Physiological measures, specifically curve differences, occasionally assume negative values. In this case, a relative expression of the difference as in Equation (1) can be misleading. Thus, absolute values were used to describe the physiological differences between copy and dual tasks (obtained by a simple subtraction). Both performance and physiological metrics were subjected to a paired *t*-test (pre-post) to test whether, after training, more attentional resources were available for the BCI task to compensate for the increased workload.

Secondly, a power model was fit to the BCI accuracy across the five sessions to test if the development of performance would be congruent with Logan’s *power law of practice*. Finally, the NASA-TLX was analyzed with a focus on the *mental demand* subscore, such that a potential workload decrease (indicating more attentional resources available) could be observed.

### Intensity discrimination task (session 1 and 5 only)

To test whether performance increases could be due to improved tactile sensitivity, a simple forced-choice intensity discrimination task was applied in the first and last session (before the EEG preparation). Here, participants received an array of two-stimulus trials. Each trial was announced by a short audio cue and consisted of two short vibrotactile stimulations (220 ms each) which were applied by the tactor device at the right knee. The first vibration was at a fixed intensity of 100%, followed by a brief pause (500 ms) and then a second vibration with an intensity that was set pseudo-randomly in 5% steps between 50% and 100%. During the task, pre-recorded tactor noises were presented over the speakers, such that the participants could not hear the sounds from the tactor devices during the tactile discrimination task, which otherwise could have helped to discriminate the stimuli auditorily.

Immediately after each trial, participants had to report whether they had perceived the intensity of the stimuli as *equal* or *unequal*, and all responses were recorded. Since this was a forced-choice approach, a trial was not repeated if a participant was unsure. Instead, when an intensity range was found in which the responses (*equal* or *unequal*) were mixed, this range was tested more frequently and extended in 5% steps to either side until the participant clearly favored one response over the other (i.e. with a ratio of at least 1:4 over several trials). Due to this dynamic approach, the total number of trials was not fixed (on average, participants performed 42.4 (*SD* = 9.5) trials per session). The participants received no feedback about the correctness of their response, since this experiment was designed purely as an assessment, not as a sensitivity training exercise.

**Figure 2:**
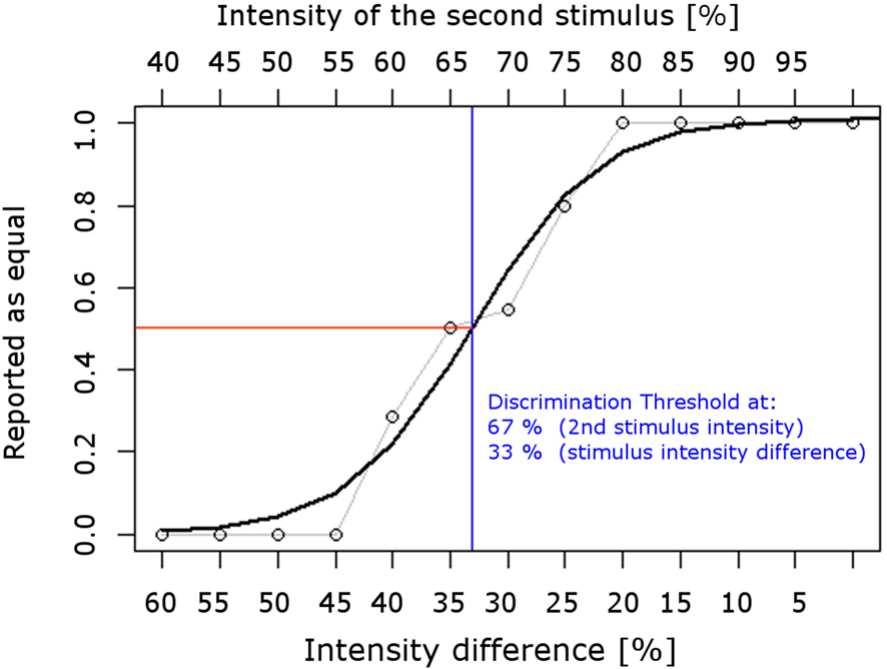
Intensity discrimination example, indicating the ratio of equal/unequal responses from one participant. Here, responses were ambiguous in the range between 40% and 25% intensity differences. We defined the intensity at which both answers would be given with the same probability (0.5) as the discrimination threshold. This point was estimated using a sigmoidal fit.

The ratio of the responses given for every intensity pair was projected onto a 0–1 scale, representing whether the stimuli were never (0) or always (1) perceived as *equal*. We defined the discrimination threshold at 0.5, at which both responses were given equally often. This point was estimated by fitting a sigmoidal function using a nonlinear least squares method over the data (see Fig. 2 for an example). In seven cases (of 58), an automatic fit was not possible because the threshold was very distinct, with values jumping from 0 to 1 without intermediate values. Thresholds for these cases were estimated manually (shown in supplementary material). Stimulus intensity differences at threshold levels were thus extracted from session one and five for all participants to test for H4a (increased sensitivity).

### General statistical analysis

BCI performance was primarily evaluated based on the online accuracies. We also report ITR, i.e. the amount of correct information transferred over time [bits/minute]. Bits (*B*) are calculated using the following equation (2) (*P* = accuracy; *N* = 4 = number of possible commands):

2 $$B={\mathrm{log}}_{2}N+ P {\mathrm{log}}_{2}P+\left(1-P\right){\mathrm{log}}_{2}\frac{(1-P)}{(N-1)}$$

The ITR is calculated by multiplying the bits with the possible number of selections per minute (SPM). With *N* = 4 and SPM = 3.0 at fixed values in the present paradigm, the ITR variability depended only on online accuracy. Thus, we focused on accuracy for analysis, but report the corresponding ITRs descriptively for better comparability with other studies.

Physiological P300 features: *Mean target amplitudes* and *mean difference between target and non-target* from Fz, Cz and Pz were extracted from averaged EEG epochs in a time window of 300–500 ms post-stimulus for every participant and session.

Training effects based on these variables were analysed with IBM SPSS 25®. ANOVAS were calculated with the factors *session number* (5 levels) and *electrode position* (3 levels, only for P300 analysis). If the assumption of sphericity was violated, Greenhouse–Geisser adjusted results are reported. Data from pre-post experiments (intensity discrimination and dual tasks) were subjected to one-tailed (directed hypotheses), paired *t*-tests. Effect sizes were described with Cohen’s *d*_*z*_ or partial Eta squared (*η*_*p*_^2^).

## Results

The five-session BCI training was completed by 31 participants. Since this study was focused on revealing factors related to training effects, only participants who could operate the BCI were considered relevant for the analysis. Consequently, two participants were excluded due to persistent BCI inefficiency. BCI efficiency was assumed only when average accuracies from the copy task were above the proposed 70% threshold^60^ in session five, or in at least three other sessions. Thus, a total of 145 BCI sessions from 29 participants (mean age 26.9 years, *SD* = 8.1, 21 female) were available for analysis.

### Physiological measures

**Figure 3:**
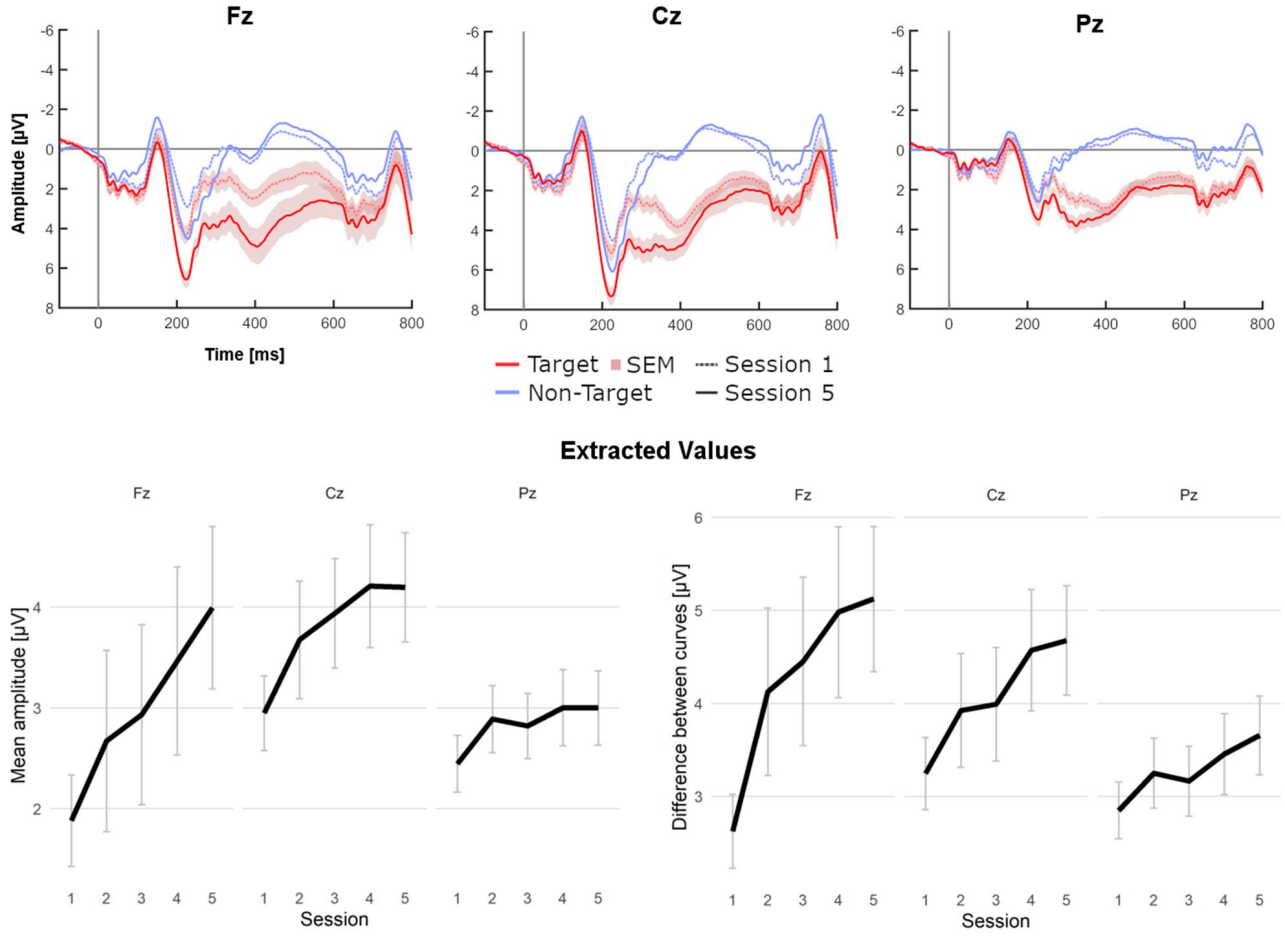
Grand average ERP data. Top: ERP shape comparison between first and last session at Fz, Cz and Pz (calibration and copy task). Bottom: Extracted mean values (amplitude & curve difference) across all sessions. Shaded areas and error bars indicate standard error of the mean (SEM).

Figure 3 (Top) shows the grand averages of target and non-target epochs at Fz, Cz and Pz (excluding the dual task), illustrating their development between the first and last training sessions. Mean amplitude and curve differences were extracted from the P300 time window for analysis and plot generation (Fig. 3, bottom). Visual analysis indicated that P300 amplitudes and differences between the curves increased substantially with training at Fz, and to a slightly lesser extent at Cz. Only minor increases were observable at Pz.

Due to a violation of the assumption of sphericity, ANOVAs were Greenhouse–Geisser corrected. Regarding the analysis of P300 amplitudes, the ANOVA revealed a significant main effect of session (*F*_(2.88, 80.64)_ = 3.27, *p* = 0.027, *η*_*p*_^2^ = 0.10). Further, we found a significant interaction (*session x electrode, F*_(2.84, 79.60)_ = 3.07, *p* = 0.035, *η*_*p*_^2^ = 0.10), but no significant main effect of electrode position.

The analysis of the difference curves (between target and non-target), revealed a significant main effect of session (*F*_(2.94, 82.30)_ = 4.15, *p* = 0.009, *η*_*p*_^2^ = 0.13). Again, the interaction effect was significant (*F*_(4.04, 113.00)_ = 3.22, *p* = 0.015, *η*_*p*_^2^ = 0.10). The main effect of electrode position was only marginally significant (*F*_(1.30, 36.49)_ = 3.72, *p* = 0.051, *η*_*p*_^2^ = 0.12).

Although the highest amplitudes and curve differences were found at Cz, *t*-tests between first and last session confirmed the most significant training effect had occurred at position Fz. Table 1 provides an overview of the extracted values and comparative statistics by session and electrode position.

**Table 1:** Statistics for physiological measures at Fz, Cz and Pz. Values extracted from grand averages of EEG data. Asterisks indicate significance.

Electrode position		Fz	Cz	Pz
Amplitudes	Mean S1 (SD) [µV]	1.88 (2.44)	2.95 (1.99)	2.45 (1.52)
	Mean S5 (SD) [µV]	3.99 (4.34)	4.20 (2.92)	3.00 (1.98)
	*t*-test (Session 5 > Session 1) [p]	0.002*	0.005*	0.062
	Cohen’s d_*z*_	0.59	0.51	0.29
Difference between Curves	Mean S1 (SD) [µV]	2.63 (2.14)	3.25 (2.09)	2.85 (1.64)
	Mean S5 (SD) [µV]	5.12 (4.20)	4.68 (3.17)	3.66 (2.28)
	*t*-test (Session 5 > Session 1) [p]	< 0.001*	0.002*	0.022*
	Cohen’s d_*z*_	0.76	0.57	0.39

Finally, visual analysis suggested a slight shift of the non-target curve toward the negative polarity across sessions, particularly at Fz in a time window of 400–600 ms. Since this was also observable in previous iterations of this paradigm^30,31^, we ran an exploratory post-hoc test, but found no significant effect (one-tailed paired *t*-test, *p* = 0.068, *d*_*z*_ = 0.284).

### BCI copy task performance

**Figure 4:**
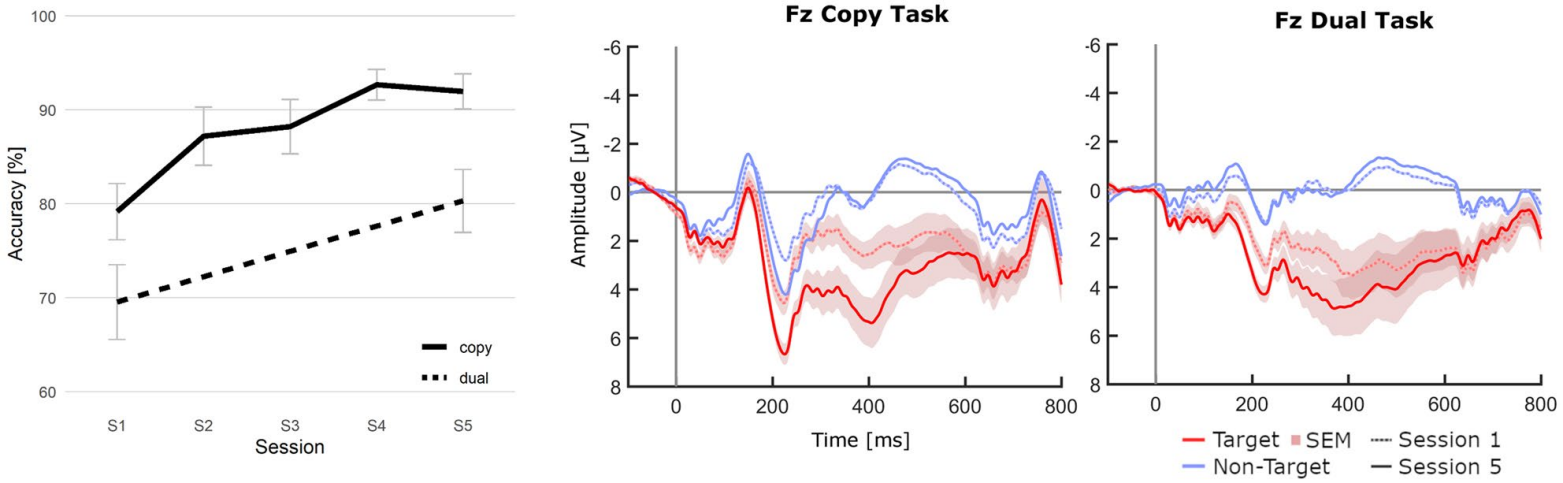
Development of BCI accuracy and comparison of copy and dual task conditions across sessions. Left: Average BCI accuracies achieved during the respective tasks. Shaded areas and error bars indicate SEM. Right: Target vs. non-target epochs at Fz from copy and dual tasks.

An ANOVA revealed a significant effect of session on the copy task accuracies (*F*_(3.10, 86.75)_ = 7.74, *p* < 0.001, *η*_*p*_^2^ = 0.22). This corresponded to an ITR increase from 3.18 (*SD* = 1.69) to 4.71 (*SD* = 1.37) bits/min. A pre-post comparison confirmed a significant increase between session one and five (*t*-test *p* < 0.001, *d*_*z*_ = 0.77), where mean accuracies increased significantly from 79.2% (*SD* = 16.1) to 92.0% (*SD* = 10.1). Accuracies across all five sessions are shown in Fig. 4.

### Dual task effects

After the dual tasks in session one and five, participants gave an average of 6.90 (session 1, *SD* = 1.88), and 7.48 (*SD* = 1.50) out of nine correct answers on details about the story, but an exploratory *t*-test revealed no significant difference. No participant had to be excluded due to a failure to pay attention to the story.

Comparing against copy task accuracies, we found significant negative effects of the dual task on BCI performance (one-tailed paired *t*-tests, Table 2). In session one, mean accuracies during the dual task significantly decreased by a factor of −0.12 (*SD* = 0.25, *p* < 0.002) to 69.5% and by a factor of −0.13 (*SD* = 0.14, *p* < 0.001) to 80.3% in session five. Notably, although both copy and dual task accuracies ranged at an overall higher level in session five, the negative effect of the dual task appeared to be relatively constant, without any significant change between sessions one and five (*t*-test, *p* > 0.05).

**Table 2:** BCI performance from copy and duals tasks and the impact of the dual task. Asterisks indicate significance.

	Copy Task	Dual Task	Comparison (copy vs. dual)	Dual Task Impact
Performance Measure	Accuracy [%]	Accuracy [%]	*t*-test [*p*]	Relative [Factor]
Session 1 (SD)	79.2 (16.1)	69.5 (21.5)	0.002*	−0.12 (SD = 0.26)
Session 5 (SD)	92.0 (10.1)	80.3 (18.1)	< 0.001*	−0.13 (SD = 0.14)

Physiological Measure	Fz Amplitude [µV]	Fz Amplitude [µV]	*t*-test [*p*]	Absolute [µV]
Session 1 (SD)	3.03 (3.03)	3.98 (4.79)	0.97	+ 0.95 (SD = 2.69)
Session 5 (SD)	5.65 (4.67)	5.70 (6.86)	0.52	+ 0.05 (SD = 4.31)

Figure 4 provides a direct comparison of averaged ERP epochs during the copy and dual tasks. We focused this analysis on the ERP difference between curves for Fz, since the most significant training effect was observed at this position. On average, the curve differences in the dual task were higher in session one (by 0.95 µV, *SD* = 2. 69) than in session five (by 0.05 µV, *SD* = 4.31), although this observation was not significant. Again, no significant training effect on this metric was revealed between the sessions. An overview of the descriptive ERP measures during copy and duals tasks, and the impact of the dual task condition are presented in Table 2.

Visual analysis suggested an asymptotic trend congruent with the *power law of practice*^55^. To further test hypothesis H3d, we calculated a non-linear regression that significantly fit the curve to a power model (*F*_(1, 143)_ = 16.08, *R*^*2*^ = 0.10, *p* < 0.001).

### Workload

The average NASA-TLX total score across all sessions was 54.17. Although the questionnaire was filled in every session, we focused analysis on sessions one and five, since intermediate sessions were lacking the potentially workload-intensive dual and discrimination tasks and are, thus, not fully comparable. All NASA-TLX scores are summarized in Table 3*,* illustrating that the scores were indeed highest in session one and five.

**Table 3:** NASA-TLX scores. MD = mental demands. PD = physical demands. TD = temporal demands. P = performance. E = effort. F = frustration. Total = global NASA-TLX score. Raw = unweighted global score.

Session	MD	PD	TD	P	E	F	Total	Raw
1	23.10	0.63	5.74	7.98	18.67	6.62	62.84	50.29
2	18.04	1.31	4.65	6.11	15.69	5.26	51.16	40.92
3	16.45	1.75	6.10	6.03	13.51	5.15	49.08	39.11
4	16.57	1.69	5.39	6.90	14.37	4.08	49.08	38.28
5	19.41	1.54	5.80	9.88	15.09	6.87	58.69	46.61
Mean	18.71	1.39	5.54	7.38	15.47	5.60	54.17	43.04

Total scores decreased from 62.84 to 58.69 with training, but a pre-post comparison did not confirm a significant effect (one-tailed paired *t*-test, *p* > 0.05). Descriptively however, there was a gradual decrease across the first four sessions, before the score increased again in session five. The highest rated subscore, *mental demand,* was of particular interest as a potential indicator for automatization, but no correlation with BCI accuracy was found (Pearson, *p* > 0.05). Still, a one-tailed paired *t*-test revealed a significant decrease of the reported mental demand with training (*p* = 0.017, *d*_*z*_ = 0.41).

### Intensity discrimination task

Discrimination thresholds were determined for each participant based on the intensity discrimination task in session one and five. Data from one participant could not be interpreted because no consensus of either equal or unequal responses was found within the tested intensity range and was hence excluded from this analysis.

**Figure 5:**
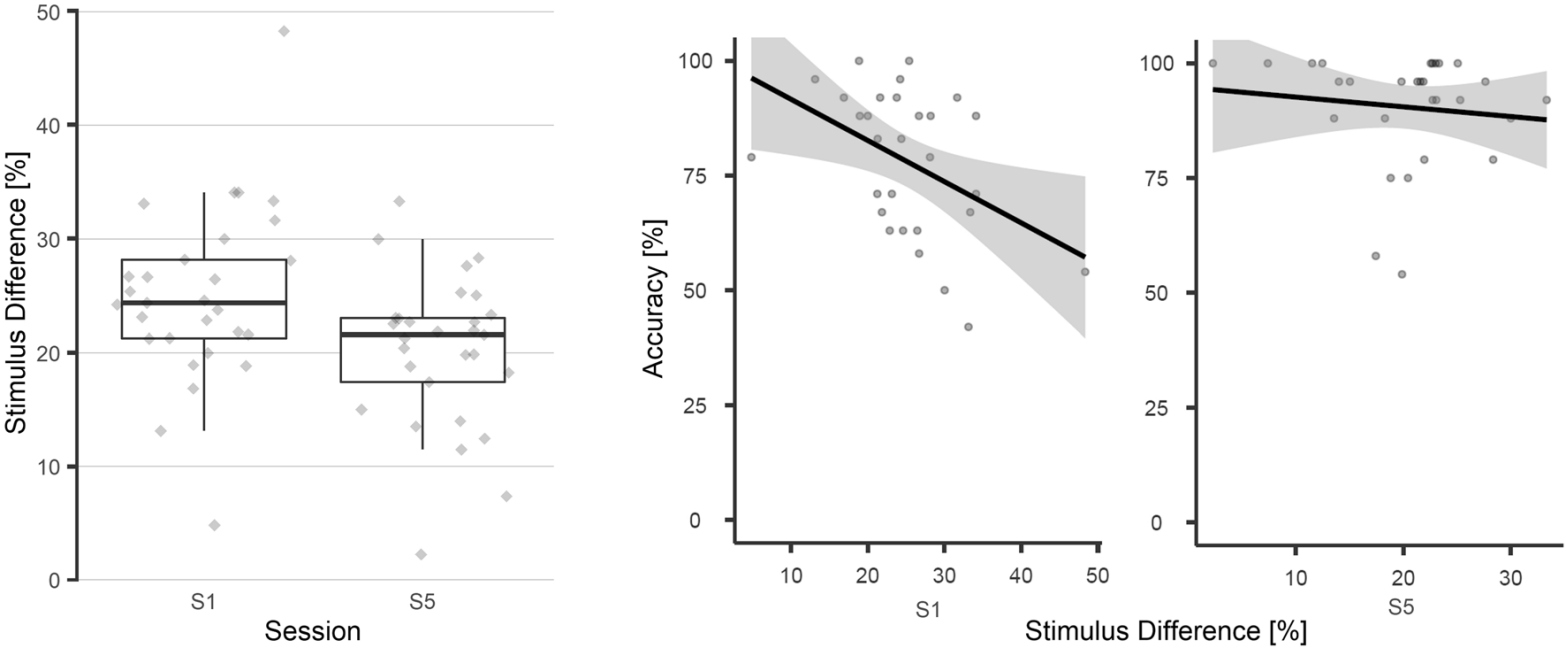
Analysis of the somatosensory sensitivity. After training, participants were able to discriminate between smaller stimulus differences, indicated by significantly lower sensory thresholds. There was a negative correlation between the sensory thresholds and accuracy in session one, but not session five.

In session five, 22 of 28 included participants were able to discriminate the vibrotactile stimuli better than before training. On a group level, we found that our participants initially required an intensity difference of *M* = 25.11% (*SD* = 7.97) to reach the threshold level, but this improved to only *M* = 20.16% (*SD* = 6.77) with training. Participants thus were able to discriminate between smaller differences of stimulus intensities. A one-tailed paired *t*-test confirmed this training effect between sessions (*p* < 0.001, *d*_*z*_ = 0.83). Figure 5 shows the distribution of individual threshold levels from both sessions and the relation between accuracy and threshold levels.

Guided by hypothesis H4b, we calculated Pearson correlations between the discrimination thresholds and copy task accuracies (Fig. 5, right) and found a significant result for the first session (*r*_*p*_ = −0.453, *p* = 0.015). Notably however, this correlation disappeared in the last session (*r*_*p*_ = −0.151, *p* = 0.442). We explored other potential correlations of the sensory thresholds, specifically between P300 amplitudes and curve differences, including the relative changes between sessions of thresholds and accuracies, but discovered no significant results.

## Discussion

### Physiological measures (H1a/H1b)

Analysis of the EEG physiology revealed a significant training effect on the mean P300 amplitude and a significant effect on the mean curve difference, confirming our hypotheses. This training effect was further substantiated by the significant increase of these measures in a pre-post comparison, especially at Fz and Cz. The relevance of these electrode positions is in apparent contrast to visual BCI paradigms, where the P300 amplitude is generally largest over parietal regions^61,62^. Still, our results are well in line with numerous previous tactile BCI studies^20,23,27,30,31,63^. It seems that in the tactile modality, the P300 is shifted toward frontal regions. Similarly, recent auditory BCI studies which implemented a five-session training program have also focused on Cz, not Pz, as the position of interest^25^. In the sample of the present study, analysis revealed that the most significant training effects had occurred at Fz (Table 1), even though mean amplitudes were highest at position Cz (both before and after training). This observation seems to further underline the relevance of frontal positions for BCI training, at least in the tactile modality.

Another particularity of this paradigm might be a possible indirect training effect on the non-target curves, again specifically at frontal sites. As in our previous study^30^, the strongest evidence for training effects on the EEG physiology was provided by the significant increase of the difference between curves. Notably, this measure differs from the P300 amplitudes because it also accounts for values of the non-target curve. Indeed, in this tactile paradigm, visual analysis repeatedly suggested a shift of the non-target curve into the negative polarity, i.e. farther away from the target curve, between the first and last training sessions (Fig. 3)^30,31^. Statistically however, this could not be sufficiently established as we found only a trend in this direction.

A possible explanation of this observation might be that in the first sessions, participants could not reliably ignore the highly salient and sometimes startling stimuli at non-target positions, causing unintended P300-like components in the EEG which contaminated the non-target epochs. While non-target stimuli can be ignored with relative ease in visual paradigms by directing the gaze on targets only, such a prefiltering of sensation is not possible in the somatosensory modality.

Indeed, there are several auditory ERP studies which report that obtrusive stimuli could evoke a P3b-like deflection even when the attention had been directed away^64,65^. Additionally, Comerchero and Polich found that the non-target amplitudes of their visual and auditory paradigm were more positive when the discrimination between target and non-target stimuli was difficult^38^. They found that the P3a deflection was enlarged at frontal electrode sites, which appears to be congruent with our own impression of a positive shift of non-target curves at Fz in the first session (Fig. 3). With continued training however, participants may have learned to better ignore non-targets by means of active suppression or habituation, which could explain how non-target epochs became more negative with time.

### BCI performance (copy task, H2)

Mirroring the observations from the analysis of ERP physiology, the accuracies of the copy task increased significantly across sessions, supporting hypothesis H2. Together with the results from previous studies, this provided strong evidence for considerable training effects in the tactile paradigm.

Average values from all sessions were significantly above the chance level^66^. In session five, our accuracies of *M* = 92.0% (4.71 bits/min) were about on the same level as those in the study by Herweg et al., who reported post-training values of 92.6% and 4.98 bits/min. However, once the authors reduced the number of stimulations per selection (and thus selection time) based on the participants’ individual abilities, a peak performance for tactile paradigms was achieved (*M* = 95,56%, 20.73 bits/min)^31^. Such optimization for speed was not done in the present study, as we put a strict focus on training factors. All relevant parameters such as the number of stimuli were thus kept identical between sessions and participants. Still, average accuracies above 90% were among the highest of tactile BCIs.

Unlike in the auditory BCI training study by Baykara et al., we found no obvious saturation of the accuracy increases after three sessions^24^. There was a minor decrease between session four and five, which might indicate the beginning of an asymptotic trend. However, this could also be explained by the fact that session five comprised additional tasks (dual and discrimination), which likely has increased the overall strain on the participants, resulting in slightly lower accuracies.

### Workload effects (H3a/H3b)

With an average NASA-TLX total score of 54.17, the reported subjective workload falls into the 60% percentile according to a meta-study^67^, so the paradigm appears to be neither particularly easy nor difficult in comparison with the various other tasks assessed with this questionnaire. In relation to non-visual BCI studies, workload was roughly on the same level. Käthner et al. for instance, reported a mean score of 57.5 of their auditory BCI^19^. However, the same participants rated the workload of a visual BCI much lower (*M* = 36.1). This confirms that the tactile paradigm, and non-visual BCIs in general, are more challenging to operate.

Notably, using the paradigm for virtual wheelchair navigation in our last study^30^ resulted in a considerably higher workload of 63.2 (80% percentile). This might either be due to a sampling effect, or the task of navigating the 3D environment was indeed much more workload intensive.

The relatively high mental demand of the somatosensory modality (as compared to the visual) might be due to the fact that this modality it is not as important for daily life of most people, and hence much less trained for quick and precise discrimination. Additionally, some participants noted that ignoring non-target stimuli was especially difficult. As already discussed, a pre-selection of somatosensory stimuli is not possible, which may have added to the cognitive workload.

Within the five-session training program, the mean total workload scores from the first and last sessions appeared to be particularly increased. We assumed this was due to the added challenge of the sensory discrimination and dual tasks. Hence, we focused on a direct comparison between these identical sessions for analysis. Regardless, support for H3a (decreasing workload) could only be found in the significant decrease of the mental demand subdimension of the NASA-TLX, but not in the total scores. This seems to underline the importance of cognitive adaptations during BCI training and confirms our hypothesis of a workload decrease, albeit only for the mental demand subscore. Notably, a decreased mental workload could indicate attentional resources had now become available for other tasks. Finally, in contrast to our expectations, a direct correlation between the mental demand and BCI accuracy could not be demonstrated (H3b).

### Dual task impact (H3c)

The manipulation of workload with the dual task appeared to have successfully increased the overall challenge and resulted in significantly reduced BCI accuracies. However, this negative impact was not affected by training. With H3c, we had predicted that task performances would become more robust against distraction or increased workload, which should have resulted in a decreased impact of the dual task in session five. This could have indicated that more attentional resources were available for the main task, possibly due to automatization. H3c, however, was not supported by the data.

The analysis of the effect on ERP measures was similarly inconclusive. In session one, the curve difference was larger in the dual task, albeit only descriptively, which was in inconsistent with our expectation of a decrease during the workload-intensive dual task. In session five on the other hand, curve differences from copy and duals tasks were nearly identical, indicating that the dual task had little impact after training.

### Accuracy development (H3d) and automatization

Our observation of an asymptotic learning curve is well in line with the results from other tactile training studies^39^. Analysis revealed that the shape of the curve (accuracies across training sessions) was consistent with Logan’s *power law of practice*^54,55^, as predicted by hypothesis H3d. Hence, a typical effect of automatization was confirmed for this paradigm.

Overall, we confirmed that mental workload decreased across the training sessions, and that a dual task condition could affect BCI performance as reported in similar experiments^48,49^. On the other hand, the dual task experiment revealed no evidence to confirm our other predictions: We had expected that more attentional resources would be available after training, leading to a better robustness against the distraction/workload increase. This, however, could not be confirmed since no significant facilitating effects on the relative or absolute impact of the dual task were discovered.

### Somatosensory sensitivity (H4a/ H4b)

We found a significant effect on the discrimination thresholds between the first and last session. The threshold stimulus difference decreased from *M* = 25.11% to 20.16%, indicating that after several tactile BCI sessions, participants were able to discriminate between smaller intensity differences. The vibrotactile sensitivity, thus, improved with BCI training, confirming hypothesis H4a. It seems plausible that sensitivity improvements contributed to training success, since stimulus perception and discriminability are important factors for oddball paradigms^38,68^. The experimental setup, however, did not allow to test a potential causal link between increased sensitivity and accuracy.

This result is in line with several (non-BCI) studies which implemented a dedicated tactile sensitivity training^39,40,47^. These, however, used slightly different approaches to assess sensitivity, i.e. temporal, spatial or frequency discrimination, rendering a quantitative comparison difficult. Our experiment instead offers, for the first time in the context of a tactile BCI paradigm, novel and strong evidence for improving tactile intensity discrimination. It is noteworthy that in the present study, sensitivity increased although no dedicated sensitivity training schedule was implemented.

Furthermore, the significant correlation of sensitivity and accuracy in session one partially confirmed hypothesis H4b, but no such correlation was found in session five. However, this does not necessarily imply that tactile sensitivity became less important with training. Participants who at first did not perform well in the discrimination task may have improved and converged toward a common ceiling with those who did very well from the beginning. A ceiling effect in the context of tactile learning has previously been described by Reuter et al. and might be responsible for the fact that a correlation could no longer be determined in session five^47^.

The importance of somatosensory perception, specifically for tactile BCI paradigms, is further supported by a recent study. Grigoryan et al. reported that blind participants significantly outperformed their sighted control group (both *N* = 10) in a tactile P300 BCI based on stimulation with a *braille* display^46^. Blind people are often considered to be more sensitive for non-visual stimulation due to sensory compensation^69^. In their study, Grigoryan et al. did not assess the somatosensory sensitivity of their participants directly. However, they recorded braille reading speed in the blind group and found that it correlated significantly with classification accuracy. A correlation between a well-trained sensory pathway and BCI performance, thus, seems plausible.

Overall, our observations strongly suggest a crucial role of tactile sensory perception not only for the base level of BCI performance, but for the overall training success of the tactile modality in BCI. Further studies should explore whether there is a causal link between the increases of sensitivity and performance. Either way, there are important implications for the user-centered design^57,70^ of future tactile BCIs. In potential end-users with ALS, for instance, the collagen structure in the skin is altered, although it is unclear whether this affects tactile sensitivity^71^. More importantly, patients with ALS and other end-users of BCI technology are often among the elderly, and it is known that mechanoperception becomes less sensitive with increasing age^45,72^. Tactile stimulation should therefore be applied such that the stimuli are well perceivable and easy to discriminate^73^. Finally, a specific training schedule to increase tactile sensitivity may be implemented prior to training with a tactile BCI.

## Significance and conclusion

Tactile BCI training studies are still scarce, perhaps due to the considerable effort required to conduct multi-session experiments with a reasonably high number of participants. However, it is crucial to elucidate how BCI performance develops over several sessions, and what factors are important for training success, to optimize the paradigm for its eventual application in end-users.

In the present study, we confirmed that the tactile BCI is trainable and that it can be used highly effectively, without being overly workload intensive. Notably, the dual task did not drastically impair BCI performance for many participants, so the tactile paradigm may in fact be suitable for use in daily life, where similar distractions are often inevitable. Automaticity was not conclusively demonstrated, but there was some evidence that training did reduce the demand on the attentional resources, and the discovered power trend may indicate that operation of a tactile BCI might automatize with further training.

Importantly, we have identified an increase in somatosensory sensitivity as a potential factor for training success in tactile BCIs. While congruent with already existing literature on the somatosensory modality, this effect was observed for the first time in a BCI context. Moreover, tactile sensitivity appears to correlate with BCI performance for first-time users. The importance of somatosensory stimulation should therefore be considered in future paradigms. These observations should be confirmed with further research.

## Supplementary Information

Supplementary Information.

## Data Availability

Datasets are available from the corresponding author (M.E.) on request: The raw data supporting the conclusions of this manuscript will be made available by the authors, without undue reservation, to any qualified researcher.
