# Supplementary material for: Identifying potential training factors in a vibrotactile P300-BCI
Source: Sci Rep. 2022 Aug 17;12:14006. doi: 10.1038/s41598-022-18088-w (PMC9385085; doi:10.1038/s41598-022-18088-w)
Supplement: Supplementary file 1 — Supplementary Information. [file 41598_2022_18088_MOESM1_ESM.pdf]

Identifying Potential Training Factors in a Vibrotactile P300-BCI

M. Eidel[1]*, A. Kübler[1]

[1] Institute of Psychology, University of Würzburg, Würzburg, Germany

*** Correspondence:**
Matthias Eidel
Matthias.Eidel@uni-wuerzburg.de

1. Supplementary Material

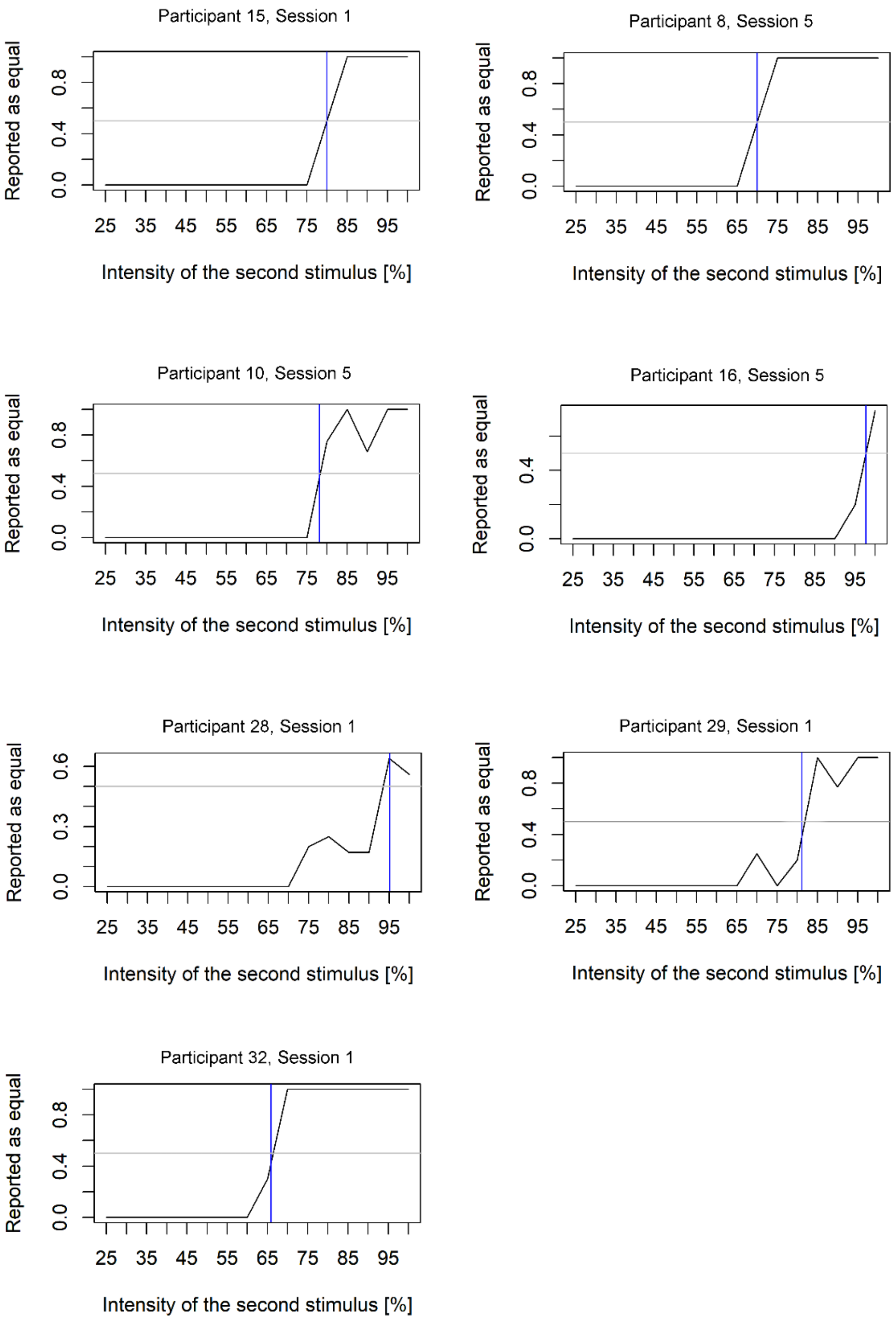

Supplementary Figure 1: *Unprocessed response curves from the discrimination task. Automatic sigmoid fit was not possible in the examples above, hence, the threshold (blue vertical) was estimated.*
